# Hypoglycaemia following upper gastrointestinal surgery: case report and review of the literature

**DOI:** 10.1186/1471-230X-10-77

**Published:** 2010-07-08

**Authors:** Brandon Bernard, Gregory A Kline, F John Service

**Affiliations:** 1 Division of Endocrinology, Faculty of Medicine, University of Calgary, Calgary, Alberta Canada; 2 Division of Endocrinology, College of Medicine, Mayo Clinic, Rochester, MN, USA

## Abstract

**Background:**

Hyperinsulinemic hypoglycemia is relatively recently recognized in persons undergoing bariatric surgery although knowledge and experience with this condition may not be commensurate with the number of such procedures being performed globally. This paper presents a novel case as an example of how such patients may present and how they may be investigated.

**Case Presentation:**

A 69-year-old man was assessed 3 months post-fundoplication surgery for postprandial hypoglycaemia with neuroglycopenia that became progressively severe. A 72-h fast failed to show hypoglycaemia. During a clinic visit, the patient became confused and had a low plasma glucose, high plasma insulin, and high plasma C-peptide; symptoms were relieved with glucose. No tumours were visualized on CT, MRI, or endoscopic ultrasound. A total body Indium111-octreotide scan was negative. Selective arterial calcium stimulation showed a high insulin gradient in the splenic and superior mesenteric arteries, suggesting diffuse pancreatic beta cell hyperplasia. The patient declined pancreatic resection and recurrent symptomatic hypoglycaemia was successfully prevented with low dose octreotide.

**Conclusions:**

Although increasingly recognized following bariatric surgery, this is the first reported development of NIPHS (non-insulinoma pancreatogenous hypoglycemia syndrome) following fundoplication surgery, as well as the first documented use of octreotide in post-operative NIPHS. Medical management may be an alternative to surgery for patients with this rare condition.

## Background

It is common to see patients presenting with symptoms attributed to "hypoglycaemia" in endocrinology and general medicine clinics. The first step in evaluation requires documentation that hypoglycaemia is in fact present and the cause of the symptoms. Once hypoglycaemia is proven, the next step is to determine whether it is mediated by insulin or by an alternate disease process. Most insulin-mediated hypoglycaemia is due to either exogenous insulin use or the presence of an insulinoma but in recent years, the differential diagnosis has expanded to include other forms of beta cell dysfunction, some of which may simulate insulinoma, as illustrated in the following case.

## Case presentation

A 69-year-old non-obese man (BMI 25.2 kg/m^2^) presented with a 3 month history of progressive symptoms of hypoglycaemia following meals. He noted that symptoms first appeared 14 years ago at which time a formal 72 hour fast failed to show any hypoglycaemia. However, his symptoms had become dramatically worse 2 months after undergoing a recent Nissan fundoplication surgery to repair a paraesophageal hiatus hernia. The episodes were characterized by agitation, sweatiness, rapid heart rate, tremor, light headedness, confusion and cognitive changes likened to alcohol intoxication that occurred an hour or two after meals. One such episode had resulted in loss of consciousness and subsequent involvement in a motor vehicle crash. There were no nocturnal symptoms and no history of weight loss, abdominal pain or diarrhoea. He denied the use of oral hypoglycaemic agents or insulin.

Initial investigations were performed during his first clinical visit when the patient was noticed to become diaphoretic and confused during the history and physical examination. The onset of these symptoms occurred at approximately 1.5 hours after breakfast. Plasma glucose was 0.8 mmol/L, serum ketones were negative, serum insulin 445.6 pmol/L and serum C-peptide of 3.54 nmol/L - values > 18 pmol/L (insulin) and >0.2 nmol/L(Cpeptide) are indicative of hyperinsulinemic hypoglycaemia. The confusion was completely relieved by oral glucose.

Computed tomography of the abdomen showed no focal abnormalities of the pancreas. Abdominal MRI showed good visualization of the pancreas with no dilatation of pancreatic ducts or focal enhancing masses in the pancreas or peripancreatic regions. Endoscopic ultrasound was negative, as was total body Indium^111^-octreotide scan.

**Figure 1:**
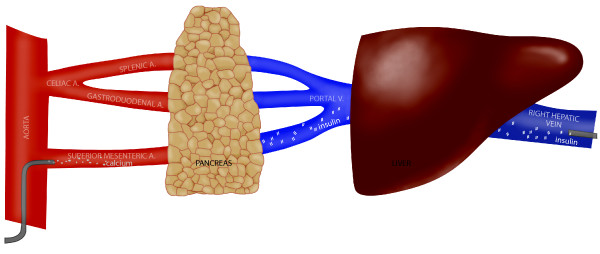
**Schematic diagram of intra-arterial calcium stimulated selective hepatic venous sampling for determination of segmental hyperinsulinemic response**.

Selective arterial catheterization and calcium stimulation testing was performed (Figure 1) with results shown in Table 1. The presence of a high calcium-stimulated insulin gradient in arteries supplying the body and tail of the pancreas suggested diffuse β-cell hyperplasia and a diagnosis of noninsulinoma pancreatogenous hypoglycaemia syndrome (NIPHS). The patient declined subtotal pancreatic resection and was successfully treated with Sandostatin 50 ug subcutaneously prior to each meal. No further significant hypoglycaemia has occurred.

**Table 1:** Insulin levels in right hepatic vein before and after sequential selective arterial catheterization and intra-arterial calcium infusion.

Sampling distribution	Pre-infusion hepatic vein insulin (mIU/ml)	Peak post infusion hepatic vein insulin (mIU/ml)	Magnitude of calcium-stimulated insulin increase
Splenic artery	3.9	62	15-fold
Gastroduodenal artery	5.1	5.4	No change
Superior mesenteric artery	6.8	31	4-fold

## Conclusions

Noninsulinoma pancreatogenous hypoglycaemia syndrome (NIPHS) was first described by Service et al. in 5 adults with symptoms of postprandial neuroglycopenia secondary to hyperinsulinemic hypoglycaemia. Diagnostic criteria for NIPHS includes a positive Whipple's triad (typical symptoms occurring in the setting of documented hypoglycaemia, relieved by glucose administration) following a mixed meal, a negative 72-h fast, negative peri-operative imaging studies for insulinoma, positive arterial calcium stimulation test, and islet hypertrophy or nesidioblastosis in pancreatic tissue [1]. This differs with the presentation of an insulinoma in that insulinomas are classically associated with fasting hyperinsulinemic hypoglycaemia and are usually identifiable on peri- or intraoperative imaging studies [2]. Diffuse calcium-stimulated hyperinsulinism from multiple segments of pancreatic vascular supply help to differentiate NIPHS from a solitary tiny insulinoma [3]. Furthermore, the neuroglycopenic symptoms of NIPHS distinguish it from "reactive" hypoglycaemia and the dumping syndrome sometimes seen in patients post-gastric bypass surgery [2,4]. No patients with NIPHS have been identified as having mutations in the Kir6.2 or SUR1 genes as occurs in certain children with a syndrome of familial hyperinsulinemic hypoglycaemia; however, the two groups do share similar histologic findings within the pancreas, namely, nesidioblastosis - a histologic diagnosis consisting of widespread β-cell hyperplasia along with diffuse proliferation and hypertrophy of islet cells from pancreatic ducts [1,2,5-7]. Recently, trophic factors and modulators of pancreatic morphology such as glucagon-like peptide 1 (GLP1) and islet neogenesis-associated protein (INGAP) have been implicated in the proposed pathologic changes leading to nesidioblastosis following upper gastrointestinal surgery [4,6,8-10]. To date, subtotal esophagectomy, subtotal gastrectomy, roux-en-Y gastric bypass, Billroth I partial gastrectomy, and Billroth II gastric bypass surgeries have all been associated with changes consistent with NIPHS, while gastric banding procedures have been shown to induce transient asymptomatic hyperinsulinemic hypoglycaemia in select patients [4,6,8,11-13]. Whether the change in gastrointestinal architecture contributes to the pathogenesis of NIPHS or whether the consequent weight loss and reduction of insulin resistance simply unmasks a primary underlying condition is still a matter of some debate [4,8,9]. In our patient's case, the onset of hypoglycaemia after the fundoplication after 14 years of quiescence suggests a causal or aggravating association but further case series would be needed to confirm such an association.

As it stands, gradient-guided surgical debulking is the only definitive treatment for NIPHS with pancreatic changes of nesidioblastosis [14]. While some have achieved desirable results with limited pancreatic resection, others have suggested that near-total or total pancreatectomy is the only definitive solution to this condition - as is the case for infants with diffuse nesidioblastosis and familial hyperinsulinemic hypoglycaemia [11,12,15,16]. Pharmacologically, symptoms have been shown to be adequately controlled with the use of diazoxide[6]. However, diazoxide use is not without potential adverse effects including hypotension and severe edema. Octreotide inhibits insulin secretion from both benign and malignant insulinomas via its effect on the G protein coupled somatostatin receptor [17]. Inhibition of insulin secretion from otherwise normal beta cells stimulated by sulfonylurea overdose demonstrates the ability of this drug to correct and prevent hypoglycaemia in beta cell hyperfunction outside of the tumour setting [18]. The present report confirms the utility of this medication to successfully treat diffuse beta cell hyperfunction/hyperplasia as well.

With the rising incidence of gastric bypass surgeries, it is important to be able to recognize the clinical picture of NIPHS and to investigate accordingly. This is the first description of the development of NIPHS following fundoplication surgery. Although octreotide has been successfully used to treat adult onset nesidioblastosis before, this is the first report of its use in post operative NIPHS [12].

## Abbreviations

NIPHS: Noninsulinoma pancreatogenous hypoglycemia syndrome.

## Competing interests

The authors declare that they have no competing interests.

## Authors' contributions

BB performed the literature review, conducted the chart review, and was responsible for the initial construction of the manuscript; GK was involved with the initial work-up and management of the patient, the diagnosis of the patient, extensive editing of the manuscript and generation of the computer graphic; FJS performed the selective arterial catheterization and calcium stimulation test at the Mayo Clinic, as well as edited the final version of the article. All authors gave final approval of the version to be published.

## Pre-publication history

The pre-publication history for this paper can be accessed here:

http://www.biomedcentral.com/1471-230X/10/77/prepub
